# Electroporation-Based Treatment Planning for Deep-Seated Tumors Based on Automatic Liver Segmentation of MRI Images

**DOI:** 10.1371/journal.pone.0069068

**Published:** 2013-08-02

**Authors:** Denis Pavliha, Maja M. Mušič, Gregor Serša, Damijan Miklavčič

**Affiliations:** 1 University of Ljubljana, Faculty of Electrical Engineering, Ljubljana, Slovenia; 2 Institute of Oncology Ljubljana, Ljubljana, Slovenia; University of California at Berkeley, United States of America

## Abstract

Electroporation is the phenomenon that occurs when a cell is exposed to a high electric field, which causes transient cell membrane permeabilization. A paramount electroporation-based application is electrochemotherapy, which is performed by delivering high-voltage electric pulses that enable the chemotherapeutic drug to more effectively destroy the tumor cells. Electrochemotherapy can be used for treating deep-seated metastases (e.g. in the liver, bone, brain, soft tissue) using variable-geometry long-needle electrodes. To treat deep-seated tumors, patient-specific treatment planning of the electroporation-based treatment is required. Treatment planning is based on generating a 3D model of the organ and target tissue subject to electroporation (i.e. tumor nodules). The generation of the 3D model is done by segmentation algorithms. We implemented and evaluated three automatic liver segmentation algorithms: region growing, adaptive threshold, and active contours (snakes). The algorithms were optimized using a seven-case dataset manually segmented by the radiologist as a training set, and finally validated using an additional four-case dataset that was previously not included in the optimization dataset. The presented results demonstrate that patient's medical images that were not included in the training set can be successfully segmented using our three algorithms. Besides electroporation-based treatments, these algorithms can be used in applications where automatic liver segmentation is required.

## Introduction

Electroporation is the phenomenon that occurs when a biological cell is exposed to an adequately high electric field, which results in the cell membrane becoming transiently permeabilized [1]. Electroporation is considered to be a universal method and platform technology since all types of cells (animal, plant, and microorganisms) can be electroporated [2]. A paramount electroporation-based application is electrochemotherapy [3], [4] which enhances chemotherapy outcome due to transient permeabilization of targeted cell membranes: because of the externally applied electric field, electroporation facilitates the chemotherapeutic drug diffusion through the plasma membrane into the cells, which would be otherwise hampered, because of the impaired or slow transport of the chemotherapeutics that are used in electrochemotherapy [5].

Electrochemotherapy is performed by high-voltage electric pulses delivery using applicators (i.e. electrodes) that are in contact with (or located near the) target tissue. Electrochemotherapy has already been introduced into clinical use for treating skin melanoma using plate or needle electrodes with a fixed geometry; the use of such electrodes imposes following predefined standard operating procedures for a successful treatment [6], [7]. Recently, however, electrochemotherapy has been introduced to clinical trials for treating deep-seated metastases in liver [8], bone [9], brain [10]–[12], and soft tissue [13]. Electrochemotherapy of deep-seated tumors imposes the use of variable-geometry long-needle electrodes introduced either percutaneously or during open-surgery [13]. Hence, only following the standard operating procedures cannot ensure success of the treatment, and patient-specific treatment planning is required for effective electrochemotherapy of deep-seated tumors [14].

Another important electroporation-based application is termed *non-thermal irreversible electroporation* (N-TIRE) and is used for tissue ablation performed using an externally applied electric field with electric field strengths and higher number of pulses than the values used for electrochemotherapy [15]. Nonetheless, the procedure is technologically very similar to electrochemotherapy and, also, requires patient-specific treatment planning [16].

To prepare a robust treatment plan for electroporation-based treatments, an anatomical model that is built from medical images (Magnetic Resonance Imaging – MRI) needs to be constructed first [17]. Construction of such a model is based on the acquisition of the patient's medical images and relies on processing the images in order to perform relevant-tissue extraction (i.e. image segmentation) [18]. Image segmentation, then, serves as the basis for generating a three-dimensional model consisting of the relevant healthy tissue (e.g. liver) and pathological tissue (i.e. tumors) [19]. Vessels may also be segmented and included into the model [20] since vessel positions have to be taken into account when defining electrodes' entry direction and relative positions. Then, a Finite-Element Model (FEM) is built and using the defined electrode parameters (number, dimensions, position), the distribution of the electrical field is calculated and optimized [21], [22] and finally presented to the attending physician.

In order to establish the concept of electroporation-based treatment planning, we follow radiotherapy treatment planning as the basis [23] using parallelisms and similarities between the planning procedures [14]. Since development of a user-friendly treatment planning would simplify electroporation-based preoperative procedures, we opt towards developing treatment planning software that will not require any prior engineering knowledge from its end-user (e.g. the attending physician). The whole treatment planning software needs to perform as automatically as possible, i.e. with minimum of interaction by the clinician, and the most challenging task is development and implementation of an automatic image segmentation algorithm. Within the clinical study of electrochemotherapy of colorectal metastases in the liver [8], we developed treatment planning procedure that includes liver segmentation. After implementing the segmentation algorithms and concluding the segmentation procedures, the latter were additionally modified using optimization results obtained using a training set of seven cases that were previously manually segmented by a radiologist. Finally, additional four cases were manually segmented by a radiologist and used for the final validation of the segmentation procedures.

In this study, we evaluated three different liver segmentation algorithms that can be used for electroporation-based treatment planning: region growing, adaptive threshold and active contours. Region growing was selected for evaluation because despite its simplicity (i.e. segments are included solely based on their intensities) this algorithm is robust and can provide good results if its basic drawbacks (e.g. oversegmentation due to leakage) are eliminated [24] using a postprocessor. Our implementation of the adaptive threshold algorithm was evaluated because this algorithm is based on a physical property, i.e. continuity of the tissue: segments on two neighboring slices are expected to be minimally different, which although being an intuitive solution which can be used as initialization of other segmentation methods [25], it proved to be accurate enough to be used as a standalone method for liver segmentation. Finally, the active contours algorithm [26] based on the Gradient Vector Flow (GVF) [27] was evaluated because of its insensitivity for intensity-based anomalies (e.g. inhomogeneity, or thin bonds connecting different segments such as the liver and e.g. kidneys) and possibility of influencing the movement of the contour by balancing the coefficient that influence attraction of the contours by the image or by the contour's inner properties. All three segmentation algorithms were optimized on a training set of seven cases, i.e. quantitatively assessed using real case data obtained from a radiologist. Finally, algorithms were validated using additional four real cases obtained from a radiologist, therefore accuracy of how their results are produced is known.

## Methodology

### 2.1. Automatic Liver Segmentation

#### 2.1.1. Importing DICOM Images

The segmentation procedure begins with importing the patient's images into the treatment planning software. The latter is a MATLAB application, developed in MATLAB R2012a (Mathworks, Nantick, MA, USA) using the *Image Processing Toolbox* and *Parallel Processing Toolbox*. The procedure for loading images loads all DICOM (Digital Imaging and Communications in Medicine) [28] files from the user-defined folder, and reads their DICOM headers' *SeriesNumber* parameter in order to determine the number of different acquisition series present in the folder. Then, an image from every series is presented to the end-user (e.g. the attending physician); an image from every acquisition series is displayed and labeled using the original label that was stored at acquisition time and is read from the DICOM header as the string stored in the *SeriesDescription* parameter. Finally, the end-user determines which acquisition series will be used for planning of the electroporation-based treatment.

After that, all the images from the selected acquisition series are loaded and sorted according to their spatial location (i.e. according to their Z-index which can be read from the DICOM header as the *SliceLocation* parameter) using a common bubble-sorting algorithm. If all obtained Z-indexes after bubble-sorting are not evenly distributed, empty slices are inserted where the slices are detected as missing. However, since missing slices may indicate corruption of the patient's images collection, the software does not try to interpolate the missing slices but notifies the end-user instead.

Besides the image data, essential DICOM metadata is loaded; the metadata structure appended to the slices includes these parameters: *Width, Height, SliceThickness, PixelSpacing, Modality, AcquisitionDate*, *BitsAllocated*, and Volume of Interest (VOI) parameters *WindowCenter* (WC) and *WindowWidth* (WW) which are most important. Namely, WC and WW determine how source image data need to be interpreted when displayed; therefore, an initial sigmoid transformation using parameters WC and WW needs to be performed first for the image data to be displayed correctly. The latter is done within the preprocessing procedure.

#### 2.1.2. Preprocessing

For segmentation algorithms to perform without problems, the imported slices first need to be preprocessed. Preprocessing is a procedure which is executed on each slice separately; therefore, the procedure is non-recursive and can be run in parallel using multiple processors or processor cores. Since the preprocessing procedure comprises of several steps, the steps are marked for debugging and algorithm evaluation purposes by storing partial results (i.e. partially preprocessed slices) into separate *layers*, starting with the *source* slices (i.e. slices stored as DICOM data), which enables the developers of the algorithms to have a clear overview of the whole preprocessing procedure.

Interpretation of imported (i.e. *source*) slices is defined by the Volume-of-Interest (VOI) parameters (i.e. Window Center – WC, and Window Width – WW) that are stored as DICOM metadata. Since WC and WW differ from slice to slice, each slice first needs to be transformed from the imported data values (i.e. *source* layer) to the normal values (i.e. *original* layer) which are defined by the VOI parameters. The transformation can be performed using a sigmoid function as defined by the DICOM standard [29]; the transformation is described using Equation 1.
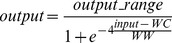
(1)


**Figure 1:**
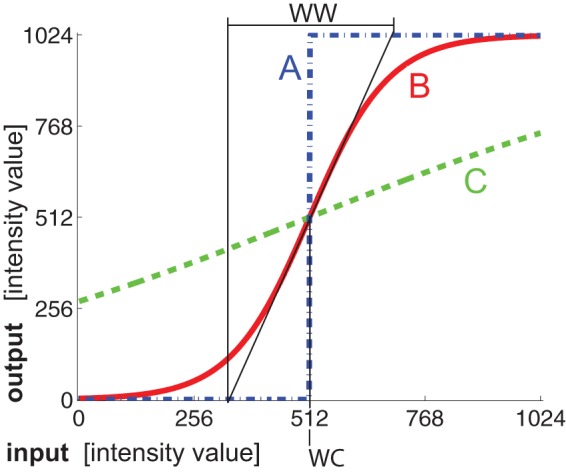
Sigmoid transformation function for preprocessing purposes. Presented is an example sigmoid function (B), with approximations of the step function (A: *WC  = 512, WW  = 1*) and linear function (C: *WC  = 512, WW  = 2048*).

As seen in Figure 1, such a transformation can be used for multiple purposes. When the WW parameter is small (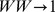), the sigmoid function (Figure 1B) changes into an approximation of a step function (Figure 1A) and can be used for thresholding, the WC parameter being the threshold value and the output value being Boolean with possible values (*0, output_range*). When the WW parameter is large (e.g. *WW > output_range*), the sigmoid function changes into an approximation of a linear function (Figure 1C) and can be used for linear transformations, the output value residing in the range (*0, output_range*) depending on the WC and WW parameter values.

After transforming the *source* slices into *original* slices using the transformation from Eq.1 and parameter values (*WC*, *WW*, and *output_range*) from the DICOM metadata, the slices are then de-biased. De-biasing is a procedure that removes intensity inhomogeneity [30] that is caused because the magnetic field in the area where the patient is positioned is not equally intense (i.e. the magnetic field is more homogeneous in the focal part of the device); a publicly available inhomogeneity correction algorithm was implemented for de-biasing [31]. Then, filtering of the slices, which is necessary for noise elimination, is performed by applying an average and a Gaussian blur filter (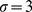), both with window sizes of 3×3 pixels. Finally, another sigmoid transformation is applied to the slices using fixed VOI parameters (*WC = 20000, WW = 100, output_range = 2^16^*) which ensures the intensity distribution of the slices is redistributed in the whole 16-bit range regardless of the *source* slices' range, and an adequate contrast which is dependent on the WW parameter. The fixed VOI values were selected based on our experience using real case data, and assure that the liver segment will have an appropriate intensity value range for the segmentation to be successful. After these procedures are applied, the resulting slices are stored as *preprocessed* slices (i.e. on a separate layer) and are ready for segmentation.

#### 2.1.3. Referential Slice

**Figure 2:**
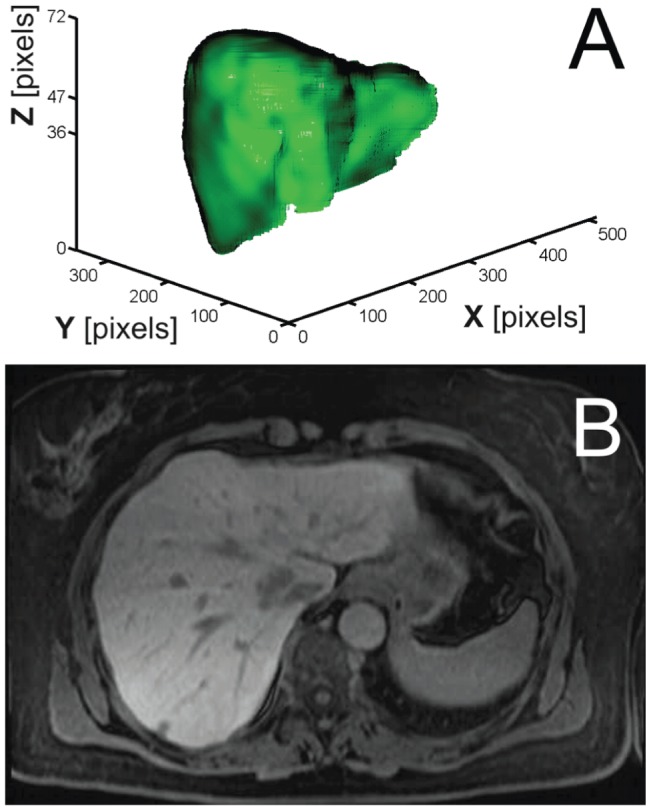
An example three-dimensional liver object. The presented object consists of 72 slices (*Z_MAX_ = 72*, A) and includes slice *Z_REF_ = 47* as the referential slice (B).

First, we define the *referential slice* as the slice in the patient's medical images collection with a high probability to include a large liver segment. We define *Z_REF_* as the index (i.e. spatial location) of the referential slice using Equation 2:
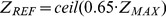
(2)where *Z_MAX_* is the number of all the slices in the patient's medical images collection and the constant 0.65 was found empirically on real case data. Using Equation 2, we have a high probability of obtaining a *referential* slice with a liver segment that is morphologically similar to the liver segment shown in Figure 2B.

The identified *referential* slice serves as the beginning point of segmentation, i.e. the slice where segmentation is initiated, and its identification is independent of the chosen segmentation algorithm. Hence, there is a high probability that the *referential* slice will include a liver segment as the one shown in Figure 2B. For region growing, the *referential* slice is used as the slice that is presented to the end-user (e.g. attending physician) in order to place the initial seed on the slice; for adaptive threshold (and also for active contours which use adaptive threshold for contour initialization), the *referential* slice is used for comparing the dynamically thresholded slice to the presets that include similar liver segments, and marking the threshold value with the highest similarity to any of the presets as the initial liver segment.

#### 2.1.4. Region Growing Segmentation Algorithm

We first implemented an intensity-based segmentation algorithm known as the region growing algorithm. The latter determines whether voxels are part of the target region or not by comparing their intensities to the intensity of the initial seed. The initial seed is a voxel manually selected by the end-user (e.g. the attending physician) at the beginning of the procedure; in our case, the *referential* slice is presented to the end-user who is required to click on the liver segment where there are no internal liver structures such as vessels or tumor nodules. The pixel clicked then serves as the initial seed voxel.

**Figure 3:**
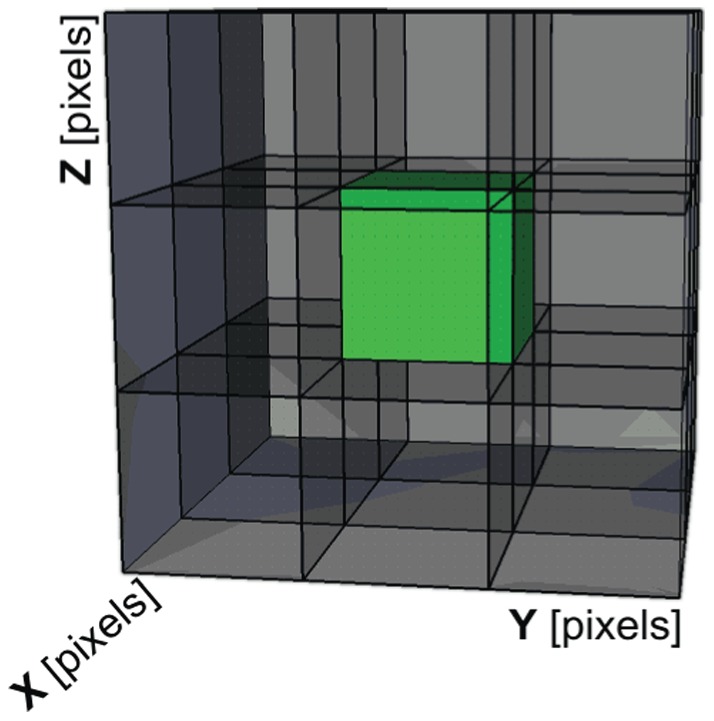
Representation of the initial seed voxel and its 26 neighbors.

The region growing segmentation algorithm works in three dimensions and evaluates the voxels that are scheduled into the queue. At the beginning, a single voxel is added to the queue, namely the initial seed. The algorithm examines the *current* voxel in the queue by comparing the intensity of every *current* voxel's neighbor to the intensity of the *current* voxel, as shown in Figure 3 where an array of 3×3×3 voxels is displayed and the middle voxel represents the *current* voxel (i.e. 26-connected neighbors).

**Figure 4:**
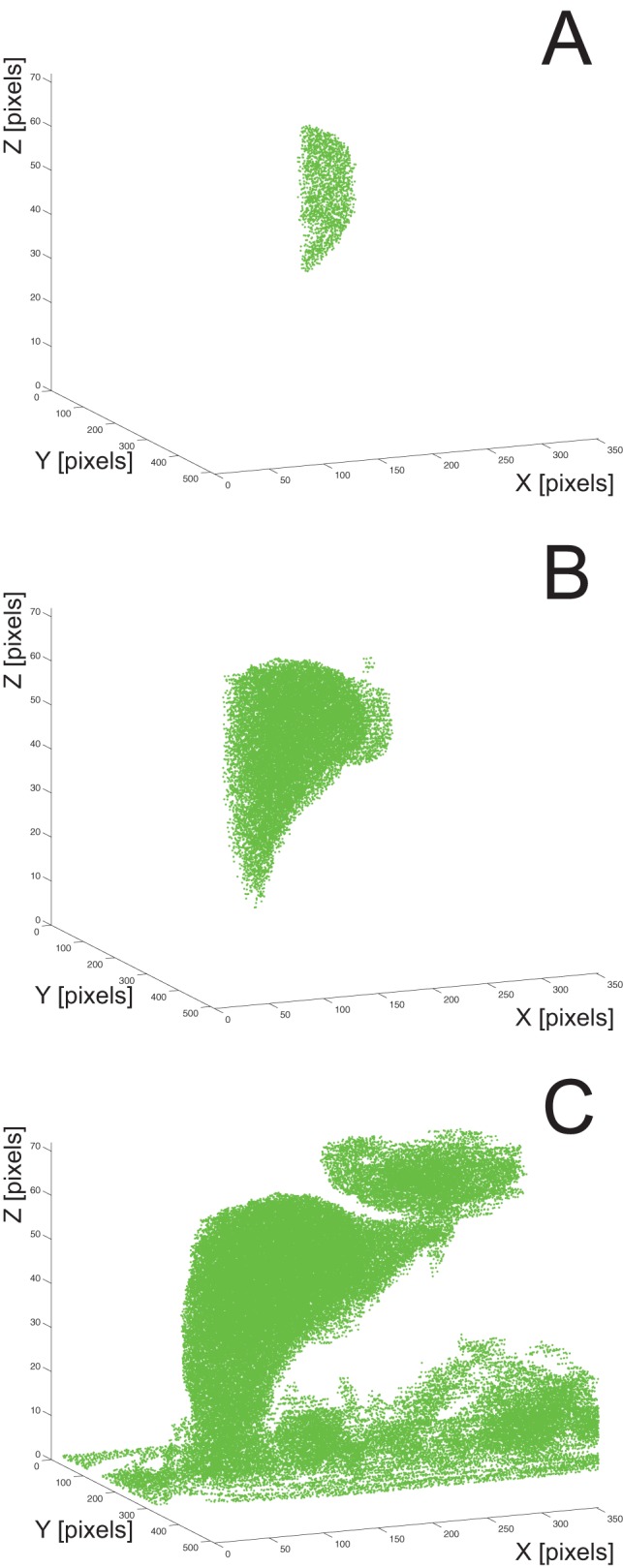
Progress of the region growing algorithm performing liver segmentation. Presented is the functioning of the algorithm after 40.000 evaluated voxels (A), after 400.000 evaluated voxels (B), and after all the voxels have been evaluated (C). The initial seed is located at *X = 192, Y = 209, Z = 47.*

Since intensity of the target region varies in all three dimensions due to inhomogeneity, it is imperative to allow some intensity deviation when evaluating if the neighboring voxels belong to the target region. The allowed intensity deviation is defined using a threshold deviation value (e.g. setting the threshold deviation value to 0.20, which is the value we used, determines the intensities that are acceptable for inclusion into the target region; the determined intensities reside in the range 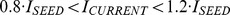, where *I* denotes the voxel's intensity and a *bit* is its unit). Therefore, any of the evaluated neighbor voxels that have the intensity in the defined range are marked as part of the target region by being added to the queue. After all the neighbors of the *current* voxel are evaluated, the algorithm evaluates the next voxel in the queue; the next voxel becomes the *current* voxel and its neighbors are evaluated. The procedure is repeated until there are no voxels left in the queue. Finally, all the voxels that are stored in the queue represent the target region which in our case is the liver. Figure 4 displays progress of the segmentation based on region growing after 40.000 evaluated voxels (A), after 400.000 evaluated voxels (B), and after all the voxels in the queue have been evaluated (C).

Due to leakage, region growing may include unwanted segments (e.g. the lower part of the heart, as seen in the upper right part of Figure 4C) which are later eliminated by the postprocessing procedure.

#### 2.1.5. Adaptive Threshold Segmentation Algorithm

**Figure 5:**
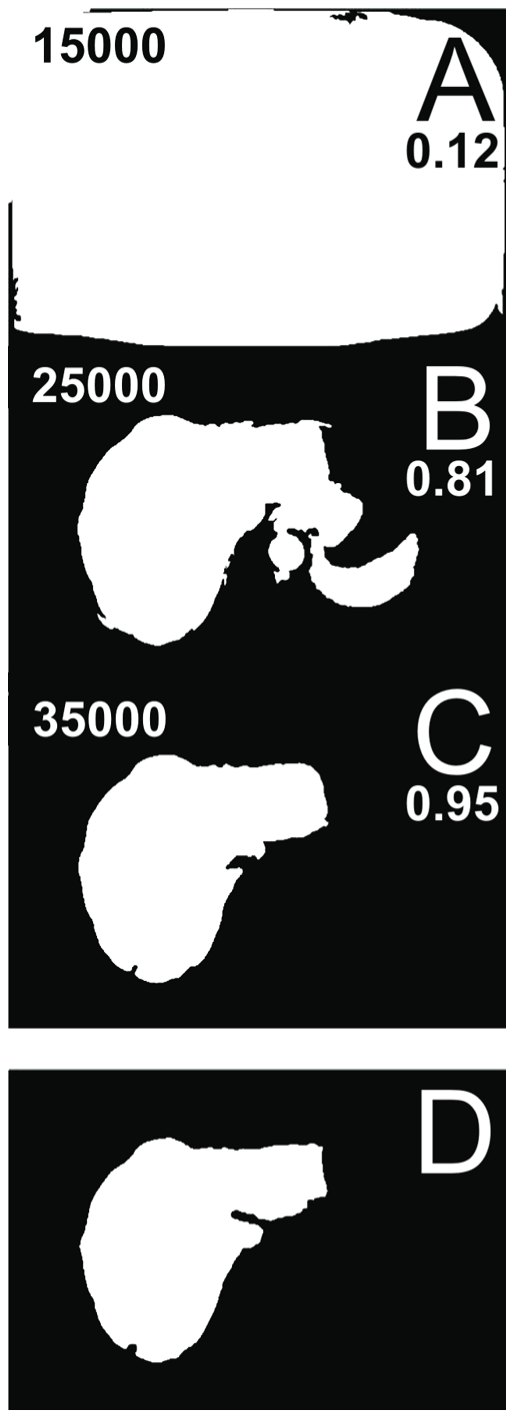
Adaptive threshold algorithm functioning. Demonstrated is the functioning of the algorithm comparing a slice while sweeping the intensity threshold value; presented are three examples where the intensity threshold value is set to 15.000 bits and similarity is 0.12 (A), 25.000 bits and similarity is 0.81 (B) and 35.000 bits and similarity is 0.95 (C); the comparison is done to the previous already-segmented slice (D).

The second liver segmentation algorithm that we evaluated is adaptive threshold algorithm. We developed this algorithm as an upgraded threshold-based algorithm that executes filtering of the current slice using a threshold function while sweeping the intensity threshold value and at the same time comparing the currently filtered slice to the previous already-segmented slice (i.e. the maximum similarity criterion). The intensity threshold value is swept over the whole intensity range, and the similarity comparison is done using normalized cross-correlation which performs segment area comparison (i.e. surface overlap). Similarity to the previous slice is chosen as the criterion for segment determination of adaptive threshold algorithm because the difference in liver shape and size between two neighboring slices is expected to be minimal; therefore, choosing similarity with the previous properly segmented slice as the criterion ensures that the current slice will also be segmented properly. Hence, the error of such a procedure is cumulative and shall the segmentation fail on one slice, all the following slices will be improperly segmented as well. The procedure is shown in Figure 5, where the current slice with three different threshold values (Figures 5A, 5B, and 5C) is compared to the previous slice (Figure 5D).

**Figure 6:**
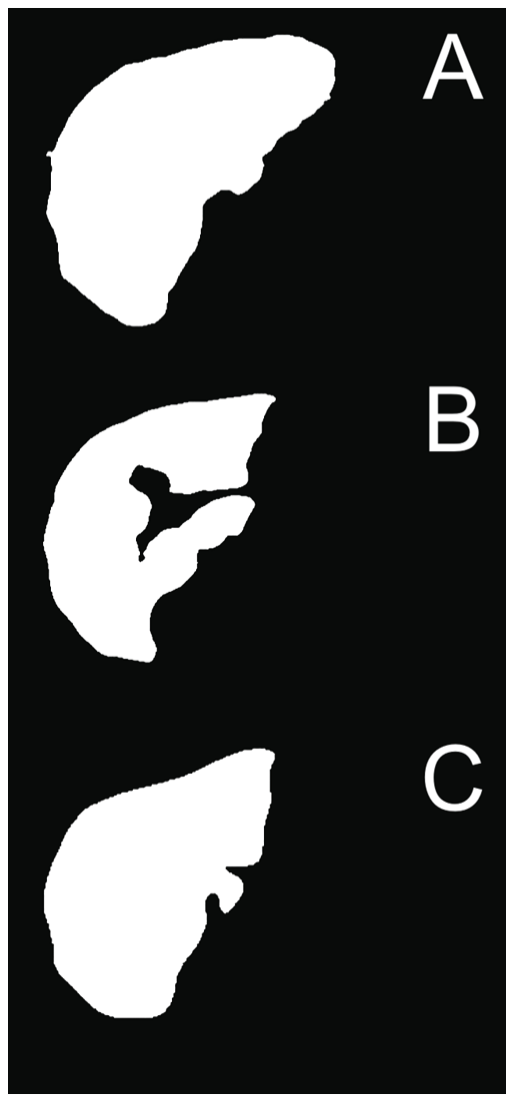
Three example liver presets used by the adaptive threshold algorithm. The preset are used for determining the final intensity threshold value of the *referential* slice.

The comparison results (i.e. similarity) which are obtained using normalized cross-correlation are stored during sweep for each intensity threshold value. After the intensity threshold value sweep is done, the intensity threshold value with the highest similarity (i.e. the maximum normalized cross-correlation) is selected, and the current slice is, finally, transformed using the selected intensity threshold value. The procedure is started from the *referential* slice and repeated on the following slices until the end of the slices collection; then, the procedure is restarted from the *referential* slice to the beginning of the slices collection. If the highest similarity is lower than a certain similarity threshold, the algorithms determines the segment has ended and empties the current and all the following (or previous, depending on the segmentation Z-direction) slices; in our case, the similarity threshold was set to 0.70 based on our experience on real case data. Moreover, since the *referential* slice has no prior slices it could be compared to, a set of six presets that include various possible liver segments is used instead, and the maximum similarity to any of the presets indicates the final intensity threshold value for the *referential* slice. Three out of six example presets that are used for thresholding the *referential* slice are shown in Figure 6.

#### 2.1.6. Active Contours Segmentation Algorithm

The third algorithm for performing liver segmentation that we evaluated for electroporation-based treatment planning is the active contours segmentation, sometimes referred to as the *snakes* segmentation algorithm. This algorithm is based on placing a deformable (i.e. active) contour, which is a closed curve made of points, on the same location as certain voxels (i.e. the initial contour position). Then, for each point of the active contour (located at the *current* voxel), the energy of the *current* and all its neighboring voxels is calculated based on four energy contributions: elasticity of the contour's point in the *current* voxel, curvature of the contour's point in the *current* voxel, magnitude of intensity-based energy in the neighboring voxels, and direction of the intensity-based energy in the *current* voxel. Each point of the active contour is, then, moved to the voxel with the lowest energy. The procedure is repeated until the active contour reaches the desired location (e.g. after a defined number of iterations).

**Figure 7:**
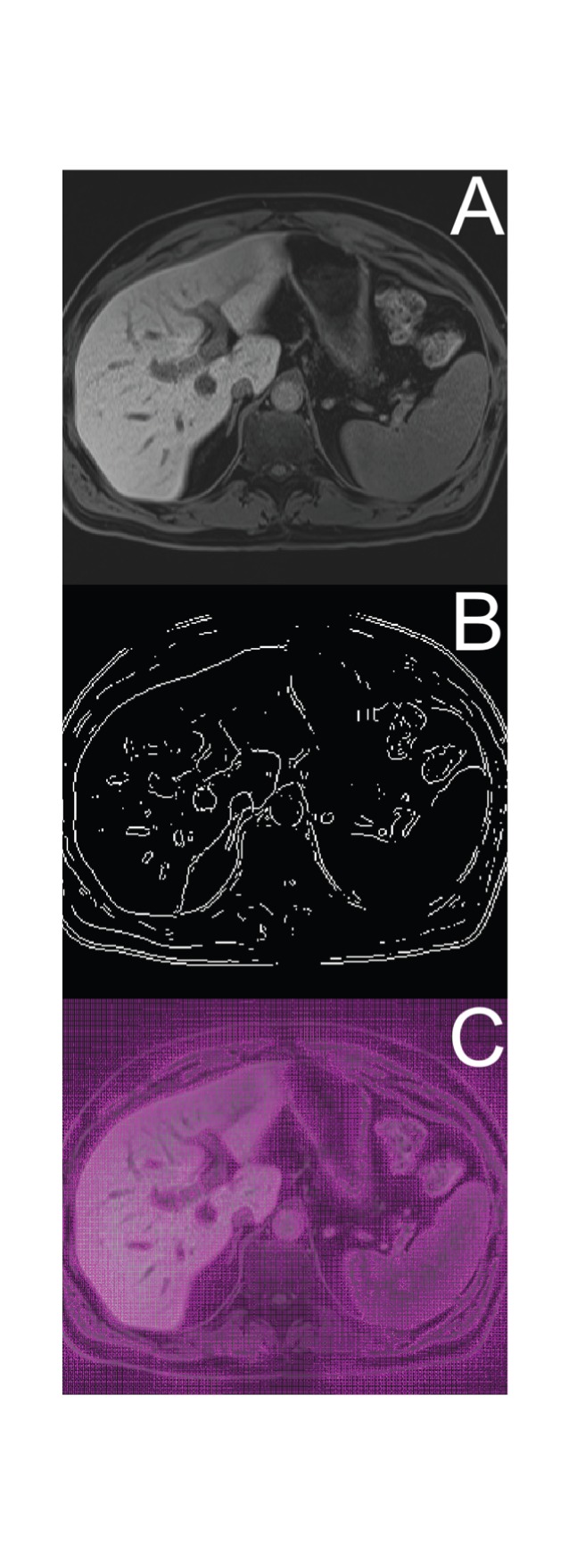
Active contours algorithm and the Gradient Vector Flow map. Presented is an example original liver slice (A) with its edge map (B) and overlaid with a calculated Gradient Vector Flow (GVF) map (C).

The active contours segmentation algorithm is initiated by placing an initial active contour on top of the *referential* slice. The active contour is attracted by the edges in the image [26]; therefore, it is imperative to initialize the active contour by placing it near the edge of the desired segment (i.e. the liver). Hence, the adaptive threshold segmentation algorithm is used to generate the initial active contour by segmenting the *referential* slice and transforming the edge of the segment identified on the *referential* slice into a closed curve with points sorted according to their location on the segment's circumference. Moreover, the number of all the points in the active contour is reduced by decimation; in our case, the number of circumference pixels between two active contour points is limited to 8. Next, the image energy is calculated as the Gradient Vector Flow (GVF) of the image; a publicly-available GVF calculation algorithm [27] has been implemented using parameter µ = 0.2 and run in 1.000 iterations. The calculation of the GVF is based on the edge map deriving from intensities in a slice; individual steps of this procedure are shown in Figure 7.

The energy contributions to the total voxel energy are, based on our experience with optimizing algorithms using real case data, balanced using the coefficients 1, 3, 9, 3 for curve elasticity, curve curvature, GVF magnitude, GVF direction, respectively. All the energy contributions are normalized to reside within the range (0,1) in order for the energy coefficients to be properly balanced. In our segmentation algorithm, the *curve elasticity* energy contribution is calculated based on Equation 3, while the *curve curvature* energy contribution is calculated based on Equation 4. The implemented GVF algorithm already ensures normalized energy results; the *GVF magnitude* energy contribution is calculated as the magnitude of the GVF vector in the evaluated voxel, while the *GVF direction* energy contribution is defined as representing low energy (0) in the neighboring voxel that is located in the direction the GVF vector of the evaluated voxel is pointing to, while all the other voxels have a high energy (1). In Equations 3 and 4, *E* denotes the energy contribution, while *P_CURR_, P_PREV_* and *P_NEXT_* denote the current, the previous and the next points of the active contour, respectively.
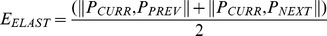
(3)

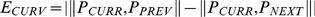
(4)


After the total energy on and around each active contour point is calculated, and balanced using energy contribution coefficients, the active contour points iteratively move toward the voxel with the lowest total energy. Since the energy depends on the active contour points' locations, the *curve elasticity* and *curve curvature* contributions are recalculated after every iteration, and the total energy is recalculated as well. The active contour movement is stopped after a predefined number of iterations (e.g. in our case, the active contour movement is limited to 100 iterations).

When the active contour movement is stopped on one slice, the procedure is repeated on another slice. The procedure is started from the *referential* slice and is repeated on the next slices until the end of the slices collection; then, the procedure is restarted from the *referential* slice to the beginning of the slices collection; therefore, the active contour segmentation algorithm may be split into two processing threads.

In order to perform segmentation using the active contours algorithm in three dimensions, the initial contour on the *current* slice is the same as the final active contour on the previous slice. Because the difference in liver shape and size between two neighboring slices is expected to be minimal on the slices where the organ that is subject to segmentation is present, the previous slice's final active contour is a good initial contour for the *current* slice. Moreover, due to the expected minimal active contour movement, less iterations are required when calculating the image's GVF since edges will attract active contours in their vicinity even when the GVF is calculated in less iterations; therefore, the processing time of the active contour segmentation algorithm is reduced (e.g. in our case, we are calculating the GVF in 1000 iterations).

#### 2.1.7. Postprocessing

After segmentation, a postprocessing algorithm needs to be executed to eliminate possible anomalies that may occur during segmentation (e.g. segment leakage). Postprocessing eliminates redundant segments that cannot be part of the final results; elimination is based on comparing neighboring slices in the direction of the third dimension (i.e. component Z) using normalized cross-correlation.

The postprocessing algorithm is initiated on the *referential* slice; namely, the probability that a slice includes only one identified segment is the highest on the *referential* slice, since segmentation was initiated on this slice regardless of the chosen segmentation algorithm: for region growing, the seed was placed on this slice; for adaptive threshold, the comparison with presets was made on this slice and also, the active contour was initiated using the adaptive threshold segmentation algorithm on the *referential* slice as well. Shall the morphological operations during segmentation split the segment on the *referential* slice into multiple segments, the first step of postprocessing eliminates such redundant segments by only keeping the largest segment on the *referential* slice.

Then, the postprocessed *referential* slice, i.e. *Z_CURR_ = Z_REF_*, is used as the basis for performing normalized cross-correlation with the next, i.e. *Z_CURR_+1* or the previous, i.e. *Z_CURR_−1* slice, respectively. The template for the normalized cross-correlation is generated by intersecting each segment on the current slice, i.e. *Z_CURR_*, with the finally postprocessed previous, i.e. *Z_CURR_-1* or next, i.e. *Z_CURR_+1* slice, respectively (depending on the postprocessing Z-direction). Then, each segment on the current slice is compared to its corresponding template generated from its neighboring slice using normalized cross-correlation; if the result of the comparison exceeds a predefined threshold, the segment is kept on the slice, else it is discarded. In our case, we set the comparison threshold to 0.65 which was found empirically on real case data. The postprocessing procedure may be split into two processing threads, since it is symmetrically executed from *Z = Z_REF_+1* to *Z = Z_MAX_*, and from *Z = Z_REF_-1* to *Z = 1* (i.e. the postprocessing Z-direction).

Moreover, if we compared the neighboring slices only by using the whole next or previous slice as the template for comparison to the current slice (i.e. without comparing separate segments on a slice), we would be unable to extract these separate segments and determine whether they derive from the target tissue (e.g. liver) or not; therefore, such comparison enables us to keep multiple segments on a slice with the possibility of eliminating segments that are not part of the target tissue. Besides, in order to allow separation of single segments that are in fact multiple segments connected by a thin bond (possibly due to leakage), all the slices are eroded before and dilated after the postprocessing procedure using a *disk* structuring element of 3×3 pixel size.

### 2.2. Validation

#### 2.2.1. Optimization using Radiologist Data Set as a Training Set

Since segmentation algorithms are required to produce not only meaningful but also accurate results, validation of the algorithms is an imperative. In our case, validation was performed as a two-step procedure. In the first step, the algorithms were optimized using radiologist dataset as a training set, and then in the second step, the algorithms were validated after being optimized using another radiologist dataset.

**Figure 8:**
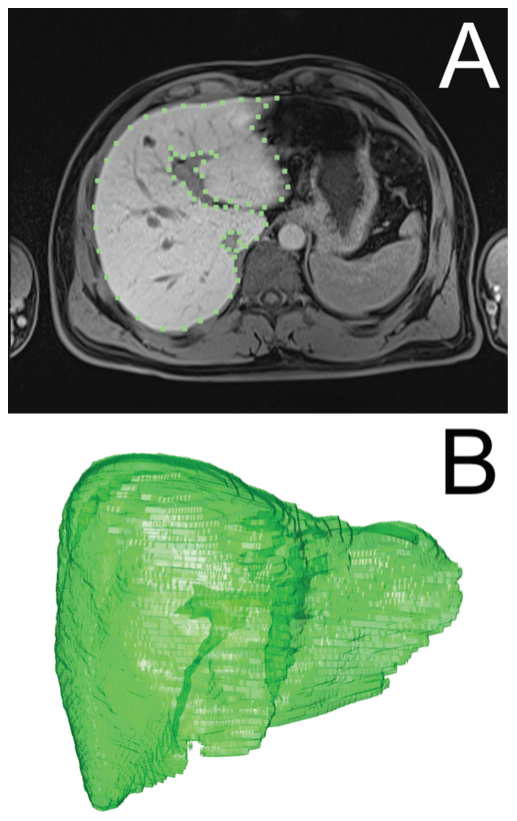
Radiologist manual segmentation procedure. Presented is a defined liver segment on a slice (A) and a final three-dimensional liver object (B).

In order to perform segmentation algorithms' optimization, seven sets of patient's liver manually segmented by a radiologist were used as a training set. The patient images that were used for development and optimization of segmentation algorithms belong to patients from the clinical study “Treatment of Liver Metastases with Electrochemotherapy (ECTJ)” (EudraCt no. 2008-008290-54, registered at Clinicaltrials.gov no. NCT01264952). The study was prospective, phase I/II, conducted at the Institute of Oncology Ljubljana, Ljubljana, Slovenia. Regulatory approvals from the Institutional board, as well as from the National Medical Ethics Committee were obtained. Written consents of the patients were obtained. Additional 4 patients were included for final validation of segmentation algorithms: these patients were under standard treatment, according to Declaration of Helsinki, and all patient data were anonymized before processing. For each segmentation algorithm, changeable parameters that significantly influence the functioning of the algorithms were defined, and their possible value ranges were defined based on our previous experience using real-case data. These changeable parameters were then subject to variation within optimization iterations; every of the seven cases that were already manually segmented by the radiologist was re-segmented using our three segmentation algorithms (i.e. region growing, adaptive threshold, and active contours) in the optimization process. During this optimization process, variation of the defined changeable parameters was performed in order to each time automatically obtain a liver object that is most similar to the one segmented by the radiologist. Figure 8 demonstrates how manual segmentation was performed by the radiologist: an example slice during manual segmentation can be seen (Figure 8A), and a final three-dimensional liver object as a result of the manual segmentation by the radiologist (Figure 8B).

After acquiring the data obtained from the radiologist and arranging them into the form that was applicable to optimization and validation (i.e. changing the data syntax by converting them to a raw format, so that inclusion into optimization algorithms was seamless), value ranges of the changeable parameters were defined. For region growing, the parameters subject to variation during optimization were the size of the noise-elimination filter mask during preprocessing, and the threshold deviation value which determines the range of the intensities that are acceptable for inclusion into the target region during segmentation (for optimization purposes, the initial seed of the region growing algorithm was chosen manually and fixed for each segmented case separately). For adaptive threshold, the parameters subject to variation during optimization were the size of the noise-elimination filter mask during preprocessing, and the initial coefficient that determines the targeted size of the referential segment (i.e. the initial liver segment on the referential slice) in the beginning of segmentation. For active contours, all four energy-contribution coefficients were varied during optimization.

Optimization was run on a workstation with Intel Core-i7 965 Extreme Edition processor (maximum frequency 3.46 GHz, 4 cores, 8 threads) with 12 GB of DDR3 memory (frequency 1.333 MHz), two 300 GB hard disk drives (velocity 10.000 rpm) running in stripe mode, and operating system Windows 7 Enterprise (64-bit). The segmented three-dimensional liver object that was produced in each iteration of optimization was compared to the corresponding model that was manually segmented by the radiologist; comparison was done on a slice-by-slice basis using normalized cross-correlation. Similarity (i.e. the results of the normalized cross-correlation) was stored together with the current values of the changeable parameters, and finally the iteration with the maximum similarity (i.e. the mean value of similarity of all slices), was chosen as the optimum (i.e. case-specific similarity, S_C_ in Table 1 and 2); the changeable parameters used in that iteration were marked as optimal parameters for the current case and currently evaluated segmentation algorithm. Moreover, optimal parameters of every evaluated segmentation algorithm were also determined for the whole evaluated series (i.e. all the seven cases) by comparing similarity of different cases with the same changeable parameters to the corresponding model that was manually segmented by the radiologist. These optimized parameters can be defined as globally-optimized parameters since their similarity (S_G_ in Tables 1 and 2) to the training set was evaluated globally (i.e. for all the cases, and not using separate parameters for each case) and is, therefore, estimated that these globally-optimized parameters are optimal for every possible liver that needs to be segmented using our algorithms.

**Table 1:** Optimization results of seven cases compared to radiologist data by mean values.

case number	REGION GROWING				ADAPTIVE THRESHOLD				ACTIVE CONTOURS			
	S_C_	std(S_C_)	S_G_	std(S_G_)	S_C_	std(S_C_)	S_G_	std(S_G_)	S_C_	std(S_C_)	S_G_	std(S_G_)
20091223	91.2%	*15.9%*	**61.1%**	*38.9%*	72.2%	*39.1%*	**72.2%**	*39.1%*	88.2%	*22.1%*	**68.8%**	*36.3%*
20100930	92.7%	*13.7%*	**86.8%**	*19.0%*	70.1%	*38.1%*	**68.1%**	*40.2%*	89.7%	*20.7%*	**64.5%**	*40.1%*
20101221	84.7%	*19.6%*	**79.9%**	*19.2%*	73.4%	*34.5%*	**70.1%**	*33.9%*	84.6%	*22.3%*	**78.6%**	*23.6%*
20110228	81.1%	*25.5%*	**65.7%**	*36.0%*	74.0%	*29.1%*	**73.2%**	*29.5%*	79.4%	*29.1%*	**67.2%**	*37.1%*
20110421	86.6%	*19.4%*	**78.2%**	*29.0%*	60.0%	*35.6%*	**40.1%**	*41.6%*	64.8%	*36.6%*	**54.8%**	*37.9%*
20110624	94.4%	*2.1%*	**94.2%**	*2.9%*	80.2%	*13.4%*	**80.2%**	*13.4%*	87.6%	*14.1%*	**67.9%**	*36.6%*
20110707	92.5%	*4.9%*	**92.5%**	*4.9%*	69.3%	*37.8%*	**69.3%**	*37.8%*	80.3%	*30.9%*	**80.3%**	*30.9%*
mean			**79.8%**	*12.7%*			**67.6%**	*12.8%*			**68.8%**	*8.6%*

Presented are mean similarities of seven cases segmented using three segmentation algorithms and compared to models that were generated by radiologist manual segmentation. Every similarity (S) is the mean value of similarities from each slice of a case, and was evaluated using individual, case-specific parameters (S_C_) or using globally-optimized parameters (S_G_). *Std* stands for standard deviation of similarities of slices within a case.

**Table 2:** Optimization results of seven cases compared to radiologist data by median values.

case number	REGION GROWING		ADAPTIVE THRESHOLD		ACTIVE CONTOURS	
	S_C_	S_G_	S_C_	S_G_	S_C_	S_G_
20091223	95.6%	**79.9%**	92.0%	**92.0%**	94.0%	**82.6%**
20100930	95.9%	**91.8%**	88.8%	**90.0%**	95.3%	**82.5%**
20101221	90.3%	**85.6%**	88.6%	**87.8%**	91.0%	**86.9%**
20110228	88.8%	**84.8%**	83.7%	**83.7%**	88.3%	**86.4%**
20110421	92.6%	**89.2%**	73.6%	**55.4%**	80.2%	**75.6%**
20110624	94.4%	**94.4%**	82.7%	**82.7%**	91.2%	**88.9%**
20110707	93.4%	**93.4%**	87.6%	**87.6%**	91.5%	**91.5%**
median		**89.2%**		**87.6%**		**86.4%**

Presented are median similarities of seven cases segmented using three segmentation algorithms and compared to models that were generated by radiologist manual segmentation. Every similarity (S) is the median value of similarities from each slice of a case, and was evaluated using individual, case-specific parameters (S_C_) or using globally-optimized parameters (S_G_).

#### 2.2.2. Final validation

After globally optimizing changeable parameters of all three evaluated segmentation algorithms, final validation was performed on additional four cases that were manually segmented by the radiologist. As opposed to the optimization procedure, the changeable parameters were fixed during validation using values obtained from optimization and previously defined as globally-optimized parameters. Then, segmentation was performed and results were compared (i.e. validation similarity, S_MN_ and S_MD_ in Table 3) to the reference model that was manually segmented by the radiologist in the same manner as when performing optimization.

**Table 3:** Validation results of four cases compared to radiologist data by mean and median values.

case number	REGION GROWING			ADAPTIVE THRESHOLD			ACTIVE CONTOURS		
	S_MN_	std(S_MN_)	S_MD_ std(S_C_)	S_MN_	std(S_MN_)	S_MD_ std(S_C_)S_MN_	S_MN_	std(S_MN_)	S_MD_ std(S_C_)S_MN_
V1	**79.4%**	*24.8%*	**87.0%**	**67.3%**	*30.5%*	**76.4%**	**54.5%**	*43.0%*	**79.5%**
V2	**87.1%**	*18.0%*	**92.5%**	**64.5%**	*35.9%*	**79.0%**	**51.7%**	*42.6%*	**70.2%**
V3	**71.3%**	*33.1%*	**85.5%**	**67.5%**	*30.1%*	**78.0%**	**72.8%**	*28.5%*	**81.9%**
V4	**49.6%**	*38.1%*	**70.5%**	**65.9%**	*35.7%*	**81.9%**	**58.2%**	*43.6%*	**83.7%**
	**71.9%**	*16.2%*	**86.3%**	**66.3%**	*1.4%*	**78.5%**	**59.3%**	*9.4%*	**80.7%**

Presented are validation results: mean and median similarities of four cases compared to manually-segmented data from the radiologist. Similarity (S) was evaluated on a slice-by-slice basis using validation-only parameters as mean (S_MN_) or median (S_MD_) of all the slices in a case. *Std* is the standard deviation of mean similarities of slices within a case.

## Results

The results of optimization, displayed as similarity to the training set, are shown in Tables 1 and 2. Both the similarity using case-specific optimal parameters (S_C_ in Tables 1 and 2) and using globally-optimized parameters (S_G_ in Tables 1 and 2) are presented. The similarity was evaluated using two possible criteria: as the mean (Table 1) or the median similarity (Table 2) of all the slices within a case of the training set.

As presented in Table 1, after being globally optimized (i.e. for all cases) our implementation of region growing algorithm provides mean slice similarities (S_G_ in Table 1) from 61.1% to 94.2% with the mean value of 79.8% (standard deviation 12.7%) which classifies the region growing as the most accurate algorithm evaluated based on the mean and also the median values of all the slices' similarities. Based on the data from Table 2, median slice similarity values for the region growing algorithm vary from 84.8% to 94.4% with the median value of 89.2%, which is the highest among all three evaluated algorithms.

Our implementation of adaptive threshold algorithm provides globally optimized (i.e. for all cases) mean slice similarities (S_G_ in Table 1) from 40.1% to 80.2% with the mean value of 67.6% (standard deviation 12.8%) which classifies the adaptive threshold algorithm as the least accurate algorithm evaluated based on the mean values of all the slices' similarities. Based on the data from Table 2, median slice similarity values for the adaptive threshold algorithm vary from 55.4% to 92.0% with the median value of 87.6%.

Our implementation of active contours algorithm provides globally optimized (i.e. for all cases) mean slice similarities (S_G_ in Table 1) from 54.8% to 80.3% with the mean value of 68.8% (standard deviation 8.6%). Based on the data from Table 2, median slice similarity values for the active contours algorithm vary from 75.6% to 91.5% with the median value of 86.4%.

The results of validation of the four additional cases manually segmented by the radiologist are shown in Table 3. The similarities are non-optimized: the segmentation results (S_V_ in Table 3) are validated using globally-optimized parameters (S_G_ in Tables 1 and 2) without further modifications of these parameters in order to show functioning of our segmentation algorithms on models which previously were not used as a training set during the optimization procedure.

**Figure 9:**
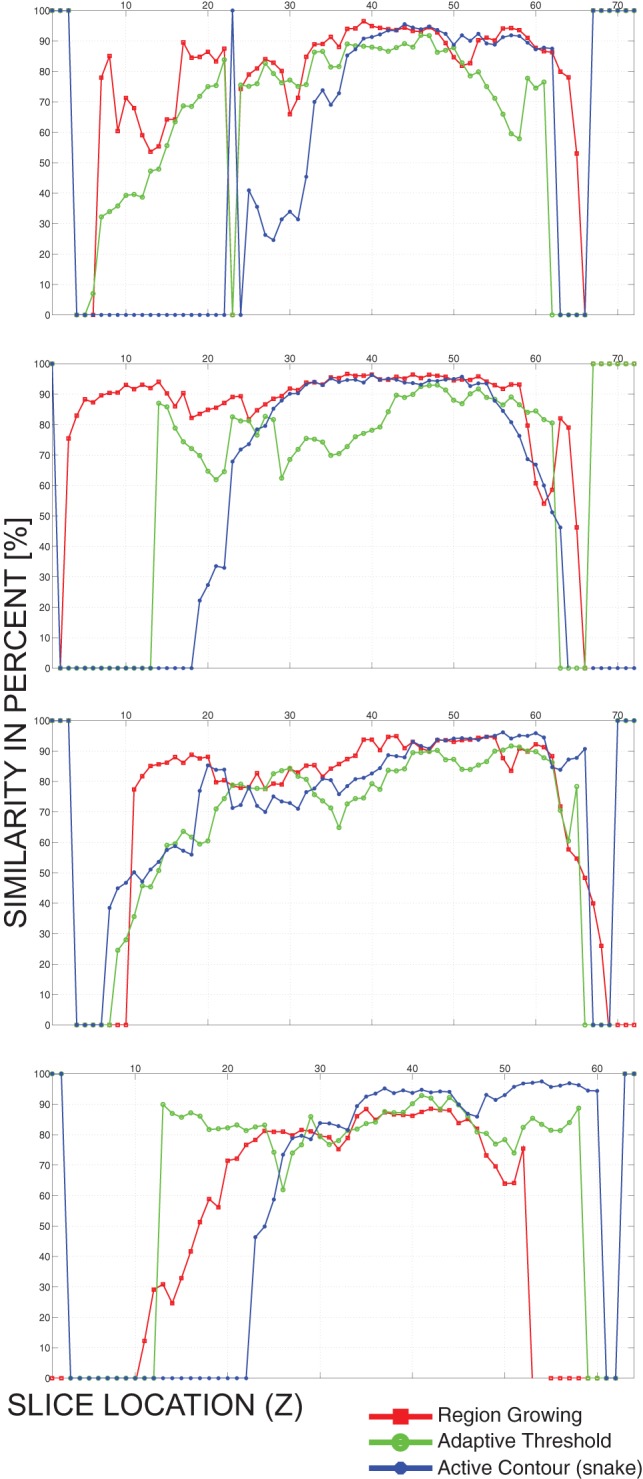
Final validation similarities of four cases (A, B, C, and D). The similarities presented are on a slice-by-slice basis, i.e. the abscissa represents the location of a slice, while the value of the graphs is the similarity of the slice with the manually validated slice.

As presented in Table 3, the region growing algorithm achieves highest similarities (mean 71.9% with standard deviation 16.2%, or median 86.3%) of the validated models. The standard deviation between different models segmented using the same algorithm is the smallest (i.e. 1.4%) using the adaptive threshold algorithm. Individual validation similarities (S_V_ in Table 3) on a slice basis are also presented in Figure 9.

Figure 9 shows that although the four validation cases (A, B, C, and D) were not included in the training set, they could be segmented using our implementations of the region growing, adaptive threshold and active contours algorithms.

## Discussion

The aim of this paper was to present three possible automatic liver segmentation algorithms and perform their evaluation by optimizing their functioning for an accurate liver segmentation; the optimization was based on seven-patients dataset segmented by the radiologist and used as a training set, while validation was performed using additional radiologist dataset consisting of four additional patients. As presented in the Results section, developing an automatic segmentation algorithm that generates accurate three-dimensional liver models is demanding, especially because of the variability of input data (i.e. liver in the patient's medical images). Nevertheless, because the region growing requires the end-user to place an initial seed (i.e. to click on the liver on one image), this segmentation algorithm cannot be classified as a fully-automatic algorithm but rather semi-automatic; however, an algorithm for automatically placing the initial seed could upgrade the region growing algorithm to a fully-automatic algorithm. The initial seed-placing algorithm can be developed based on the adaptive threshold segmentation algorithm (described in 2.1.5.) on the *referential* slice, with an additional task of selecting a pixel with a median intensity of the segment on the *referential* slice which should ensure avoidance to selecting vessels or nodules as the target region.

The liver includes or may include various inner structures (e.g. blood vessels, metastases, hemangioma, etc.) that do not have a predictable intensity range or texture; these structures may directly interfere with segmentation algorithms by possibly influencing the generation of segments. Also, certain organs near the liver (i.e. the spleen, the heart, the kidneys) have similar intensity ranges to the liver [32], which results in segmentation leakage for threshold-based segmentation methods (i.e. region growing and adaptive threshold). Moreover, active contours are attracted to edges, including the edges of the inner structures; therefore, straight-forward three-dimensional segmentation of the liver using active contours would only be possible if the three-dimensional contour (i.e. the deformable surface) was initialized entirely near the edge of the liver. Besides, the soft transitions of the organs from one slice to another often prevent detection of the organ edges, which leads to an insufficient Gradient Vector Flow (GVF) map and consequently corrupting the generated segments of the active contours algorithm. All the identified difficulties demonstrate that accurate automatic liver segmentation is indeed a challenging task. Fortunately, highest accuracy of liver segmentation is not required for electroporation-based treatment planning since tissue of interest for electric field distribution actually represents the tumor tissue, and possibly large blood vessels which must be taken into account because of electric field calculations and electrode placement. The liver tissue, however, is only a medium that surrounds the tissue of interest, i.e. the tumor that resides in the liver, since electroporation-based treatment planning is required for tumor nodules that are inside or on the edge of the liver. Therefore, the main function of the generated three-dimensional liver model besides being the medium surrounding the tissue of interest is to provide instructions to the attending physician on how the electroporation-based treatment will be performed (i.e. where the tumor is located, since it cannot be seen as it is deep-seated), and serves as electrode-insertion approximate offline navigation for the clinician.

Since similarity comparisons using normalized cross-correlation were performed on each slice of a case separately, final results of each case are presented using two representations: the mean similarity of all the slices, and the median similarity of all the slices in a case. Two representations of the data were presented since there are many individual slices with a similarity of 0%, which means whether the radiologist marked a segment on that slice and the segmentation algorithm missegmented it, or vice-versa (i.e. the segmentation algorithm detected a segment that the radiologist did not identify). Hence, such slices significantly contribute to the final results of the comparison regardless of the size of the segment that caused the 0% similarity on the slice (i.e. even a segment of only few pixels detected by the segmentation algorithms and not identified by the radiologist would produce a 0% similarity of its slice). Since such slices significantly impact the quality of the final results, the mean similarity of all the slices in a case does not reflect success of the segmentation algorithm enough. Therefore, the median similarity of the slices in a case was presented as well.

Optimization results presented in Tables 1 and 2 show that using case-specific optimization parameters (i.e. parameters optimized for every case separately, S_C_ in Tables 1 and 2), very good similarities to the training set can be obtained (median similarities of up to 95.9% using region growing, up to 80.2% using adaptive threshold, and up to 80.3% using active contours). However, results applying to case-specific parameters can only be achieved if a reference model which is part of the training set is available. Therefore, globally-optimized parameters are the parameters that give meaningful information on how accurate a segmentation algorithm is. Our implementation of region growing algorithm could be optimized to achieve an 89.2% median similarity (79.8% mean similarity with 12.7% standard deviation) to the training set, which classifies this algorithm as the most optimization-prone algorithm evaluated. The adaptive threshold algorithm could be optimized to achieve an 87.6% median similarity (67.6% mean similarity with 12.8% standard deviation), and our implementation of the active contours algorithm an 86.4% median similarity (68.8% mean similarity with 8.6% standard deviation), which shows that despite being more sophisticated, these two algorithms achieved lower results than region growing. The main reason is that region growing algorithm can be effectively optimized since the optimizable threshold-deviation parameter majorly influences segmentation (i.e. it significantly influences which voxels will be part of the final tissue segment). The adaptive threshold only has one most relevant parameter that could be optimized (i.e. the initial coefficient that determines the targeted size of the initial segment on the referential slice), which does not influence segmentation on further slices. Although the active contours algorithm includes four optimizable parameters (i.e. the four coefficients that balance energy contributions), it is almost impossible to influence the movement of the active contour (i.e. the snake). Namely, even if the four energy contributions are ideally balanced, the active contour movement needs to mostly rely on the edges in the image (i.e. the two coefficients representing GVF magnitude and directions). Therefore, if the edges of the target tissue were improperly detected after preprocessing (e.g. the target tissue does not have clearly detectable edges), the movement of the active contour will produce unwanted results.

Results of the final validation that are presented in Table 3 show that new patient images that were not part of the optimization training set can be segmented using our implementations of segmentation algorithms (region growing, adaptive threshold, and active contours). The best results are provided by the region growing algorithm (median similarity 86.3%, mean similarity 71.9% with standard deviation 16.2%), while adaptive threshold (median similarity 78.5%, mean similarity 66.3% with standard deviation 1.4%) and active contours (median similarity 80.7%, mean similarity 59.3% with standard deviation 9.4%) provide lower similarity values. As it can be seen in Figure 9, there were many slices missegmented in all four cases. Most missegmented slices were produced using the active contours algorithm, while the least standard deviation is provided using the adaptive threshold algorithm. Although such missegmented slices would negatively influence not only generation of the liver model, but also detection of the tumors, this drawback is avoided using manual validation by the attending physician, which is discussed in the following paragraph. Finally, although the region growing was expected to be the least sophisticated and accurate algorithm, it proved to be the most robust of all the evaluated algorithms, providing highest accuracy and least missegmented slices.

**Figure 10:**
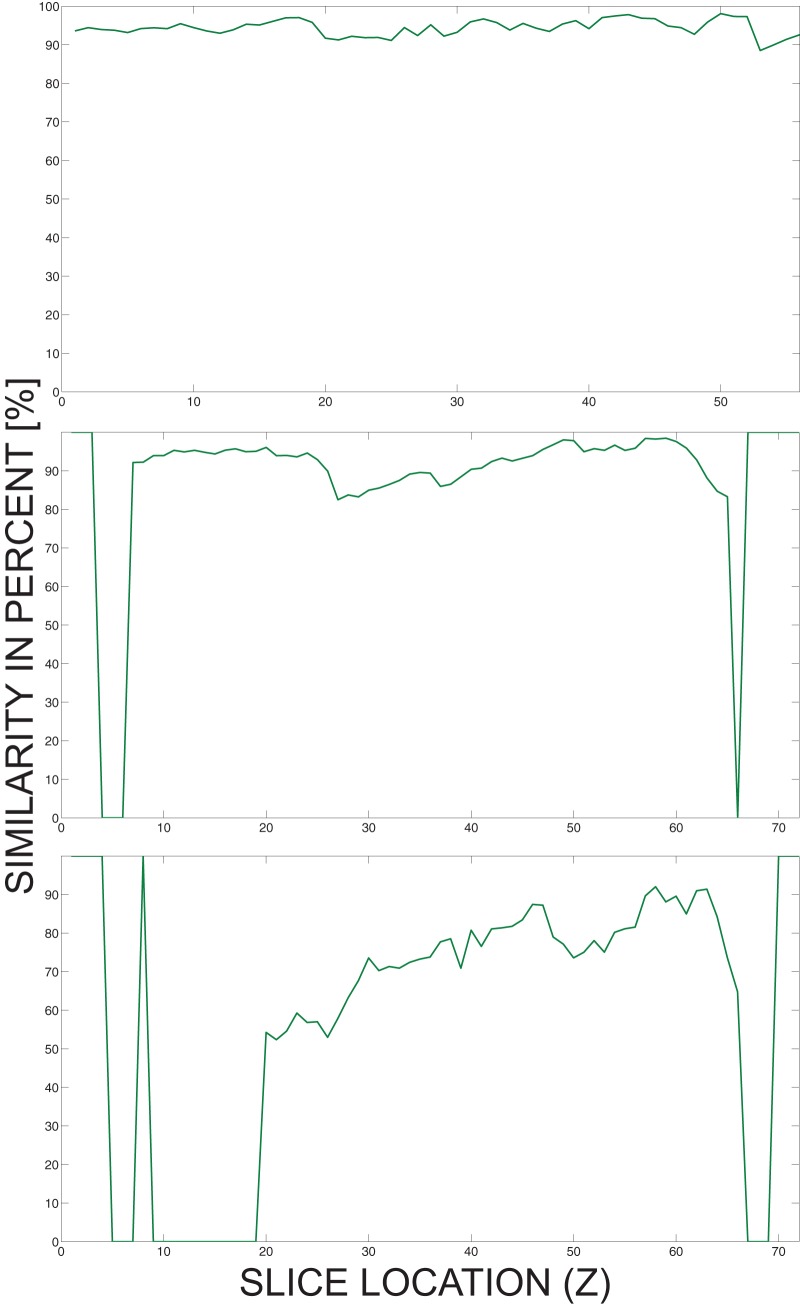
Possible miss situations when segmenting the liver. Presented are the situations where all the slices are segmented (A), three slices have been missegmented (B) or many slices have been missegmented (C).

Tumor detection from the patient's medical images is currently still in development and will be implemented as a semi-automatic procedure: identification of structures within the segmented liver (including its edge area) will be done automatically, but finally the end-user (i.e. the attending physician) will be required to manually determine which identified structures are tumors and are, therefore, subject to electroporation-based treatment. Therefore, the whole liver needs to be properly segmented, since tumors are detected as structures within or on the edge of the liver, and could be missed if some slices at the top or at the bottom of the liver are not segmented. Figure 10 demonstrates possible situations that are related to missegmenting liver segments at the beginning or at the end of the liver: Figure 10A shows an ideal case where all the slices are segmented with a high similarity to the radiologist data; in Figure 10B, there are three slices that do not include liver segments but should include them (similarity of these slices is zero); Figure 10C shows a bad case where many slices that should include liver segments do not include them. The main reason for missing segments on such slices is intensity inhomogeneity in the Z direction (i.e. not on a single slice but through slices); although the latter is actually corrected when preprocessing the images applying the sigmoid transformation using Volume of Interest (VOI) parameters from the DICOM header, the anomaly is not completely removed, especially in the beginning and in the end of the series. Since this phenomenon cannot therefore be fully avoided, and because there are rare cases that can be automatically segmented as the case from Fig 10A, manual validation of the segmented slices is required at the end of electroporation-based treatment planning procedure. The validation can be combined with the attending physician's manual identification of the structures whether they are tumors or healthy tissue, which reduces the time needed to execute the whole treatment planning procedure. Hence, the attending physician manually validates the segmented images to ensure proper liver and tumor model generation, and manually corrects them if required.

Currently, only Magnetic Resonance Imaging (MRI) images are segmented for electroporation-based treatment planning, as MRI is preferred in clinical practice for the liver. Namely, MRI is a non-invasive procedure (i.e. there is no ionizing radiation present during MRI) and using a contrast medium it can provide satisfactory images of the liver for electroporation-based treatment planning. Hence, our algorithms are currently written and optimized only for MRI images; the preprocessing procedures are prepared for expected intensity ranges and Volume-of-Interest (VOI) values that derive from MRI sources, and could therefore not perform segmentation of e.g. Computed Tomography (CT) images. However, since the modality of obtained images can be easily determined from the header of the DICOM files, and because only minor modifications of the preprocessing algorithms would be required, a modification allowing non-MRI image segmentation would not be demanding to implement.

Further steps of our research in the field of electroporation-based treatment planning include improved algorithms for tumor detection and, also, automatic vessel segmentation. Namely, segmentation of tumors and vessels needs to be also developed since algorithms for liver segmentation (i.e. region growing, adaptive threshold, and active contours) cannot be used for this purpose without thorough modifications of the algorithms. We expect to develop vessel segmentation algorithms as a combination of intensity- and morphology-based methods with the aim of extracting line-like structures from the liver.

We plan to develop electroporation-based treatment planning as a web application that will be remotely accessible from a web browser and will allow generating treatment plans by allowing the attending physician to upload the DICOM images of the patient and, after calculations and manual validation of the results, obtaining a directly applicable treatment plan. Hence, besides the automatic liver segmentation, the treatment planning software will also need to include electrode insertion and calculation of the electric field distribution with electrode position optimization in order for the treatment plan to comprise all the required information. The treatment planning procedure will be simplified in order to minimize the input of the clinician: because the segmentation of the tissue is automatic, the clinician will only need to validate the segmentation results (and if required, correct the segmentation by dragging the produced contours towards desired positions on each slice) and, finally, determine the entry direction of the electrodes. Namely, the electrodes will be automatically inserted towards the gravitational center of the tumor (or tumors), and the required number of electrodes will be proposed by the software based on the shape and size of the tumor (or tumors). Therefore, the clinician will only need to rotate the electrode array towards the excepted intraoperative entry direction, which will simplify the procedure.

Finally, since automatic liver segmentation can be implemented for other applications [32]–[34] beside electroporation-based treatments, we opt towards extending the functioning of our web-based treatment planning software for electroporation-based treatments onto related fields of surgical liver intervention planning.
